# Dynamic and quantitative assessment of blood coagulation using optical coherence elastography

**DOI:** 10.1038/srep24294

**Published:** 2016-04-19

**Authors:** Xiangqun Xu, Jiang Zhu, Zhongping Chen

**Affiliations:** 1 College of Life Sciences, Zhejiang Sci-Tech University, Hangzhou, Zhejiang 310018, China; 2 Beckman Laser Institute, University of California, Irvine, Irvine, California 92612, USA; 3 Department of Biomedical Engineering, University of California, Irvine, Irvine, California 92697, USA

## Abstract

Reliable clot diagnostic systems are needed for directing treatment in a broad spectrum of cardiovascular diseases and coagulopathy. Here, we report on non-contact measurement of elastic modulus for dynamic and quantitative assessment of whole blood coagulation using acoustic radiation force orthogonal excitation optical coherence elastography (ARFOE-OCE). In this system, acoustic radiation force (ARF) is produced by a remote ultrasonic transducer, and a shear wave induced by ARF excitation is detected by the optical coherence tomography (OCT) system. During porcine whole blood coagulation, changes in the elastic property of the clots increase the shear modulus of the sample, altering the propagating velocity of the shear wave. Consequently, dynamic blood coagulation status can be measured quantitatively by relating the velocity of the shear wave with clinically relevant coagulation metrics, including reaction time, clot formation kinetics and maximum shear modulus. The results show that the ARFOE-OCE is sensitive to the clot formation kinetics and can differentiate the elastic properties of the recalcified porcine whole blood, blood added with kaolin as an activator, and blood spiked with fibrinogen.

Coagulopathy, a condition in which blood coagulation is impaired, can cause life-threatening bleeding or thrombotic disorders resulting from a variety of conditions, including severe trauma, illness or surgery. Dynamic and quantitative assessment of coagulation is important to diagnose potential causes of hemorrhage, to guide hemostatic therapies, and to predict the risk of bleeding during consecutive surgical procedures. During surgery, trauma care and chronic disease management, clinicians often encounter the challenging task of maintaining a precarious balance between bleeding and coagulation. Most commonly, routine laboratory-based coagulation tests, e.g., prothrombin time/international normalized ratio (PT), activated partial thromboplastin time (aPTT), fibrinogen levels, and platelet numbers, are being used to assess the patient’s current coagulation status^1^. However, the tests are of limited value in perioperative and acutely ill patients because there are delays from sample transportation and centrifugation to obtaining results (45–60 min)^2^; the turnaround time is often too long for the tests to be reliable for informing blood transfusion or anti-coagulant therapy in the context of rapidly changing coagulation conditions in critically ill or injured patients^2^. Moreover, the coagulation tests are determined in plasma rather than whole blood: there is no information available on platelet function and red blood cell function.

Abnormal mechanical properties of clots are associated with the risk of thrombosis and bleeding. Thromboelastometry (TEG^R^/ROTEM^R^) has been widely used in a clinical setting for monitoring coagulation during procedures, including hepatic and cardiac surgeries, which are associated with high risk of massive bleeding^3^,^4^. However, thromboelastometry is not an ideal modality for assessing clot mechanical properties due to its poor sensitivity, repeatability, and lack of standardization^3^,^4^. While the poor standardization of thromboelastometry may be due to the absence of standard clinical protocols, it may also be related to the physical method of measurement which requires accurate measurement of stress and strain. Recently, optical methods have been developed for the assessment of the biomechanical properties of clotted blood, such as magnetomotive optical coherence elastography (MMOCE) method^5^,^6^ and laser speckle rheology (LSR) method^7^ due to the high sensitivity. MMOCE method uses fundamental resonant frequency to represent the elastic properties of clotted blood. However, it cannot monitor the dynamic change of the elastic properties during blood coagulation and needs contact between microbead and detected sample for the magnetic force application, which may change the coagulation process. The non-contact LSR method can detect the coagulation process in real-time. However, the speckle autocorrelation time constant used for characterizing the elastic properties in the LSR method cannot directly calculate the elastic modulus without subsequent calibration.

Optical coherence elastography (OCE), employing optical coherence tomography (OCT) to detect depth-resolved sample deformation, has been used to assess tissue biomechanics^8^,^9^,^10^,^11^,^12^. OCE techniques offer distinct features in comparison to ultrasound elastography and magnetic resonance elastography (MRE), including imaging spatial resolution, acquisition speed, mechanical sensitivity, and imaging penetration^10^. The spatial resolution of OCE, as set by OCT, is typically 1–10 μm: at least an order of magnitude higher than MRE and ultrasound elastography. Also, the development of OCE using phase-resolved OCT detection can assess and extract different parameters of tissue deformation with high accuracy to reconstruct the tissue biomechanical properties^8^. A recent OCE method based on shear wave measurement can provide the elastic modulus with high spatial resolution and high sensitivity^11^,^12^. The shear wave velocity *V* can be directly used to calculate the shear modulus *μ* by the following equation:


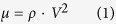


where *ρ* is the density of the tissue.

Excitation and detection are generally two characteristics of an OCE system. Various loading approaches have been proposed, such as the use of magnetomotive nanoparticles as the internal transducers for vibration^5^,^6^ and applying acoustic radiation force for remote stimulation^12^,^13^. Using an ultrasonic transducer, ARF can remotely induce a shear wave without the contact in the OCE system. An OCT Doppler variance method for measuring the transverse vibration induced by ARF orthogonal excitation (ARFOE-OCE) was recently developed^13^. The vibration is perpendicular to the OCT beam, and the shear wave propagates along the OCT beam. The shear modulus, indicating tissue stiffness, in a two-layer phantom at the position beyond the OCT imaging depth was accurately measured by the method^13^.

In this work, we report for the first time the non-contact and real-time measurement of the shear wave velocity and elastic modulus during the dynamic blood coagulation process using the ARFOE-OCE method, where the ARF orthogonal to the OCT beam produces the vibration of the blood tissue and the shear wave propagation is visualized utilizing phase-resolved OCT detection with the Doppler variance method. Doppler variance analysis works better if the vibration direction is perpendicular to the optical detection direction when compared with Doppler phase analysis^14^,^15^. We validate the feasibility of ARFOE-OCE for quantitative assessment of the dynamic porcine whole blood coagulation process by temporally tracking induced shear waves. Then we apply this method for differentiating the coagulation process of the recalcified porcine whole blood and the blood added with kaolin as an activator using coagulation metrics, including reaction time, clot formation kinetics and maximum shear modulus. Fibrinogen is a plasma protein that can be transformed into an insoluble fibrin by the action of thrombin in the final stage of the blood coagulation and plays a key role in the hemostatic system. The normal plasma fibrinogen level ranges from 2.0 to 4.5 g/L, while increased plasma fibrinogen concentration can be associated with increased blood coagulability and viscosity, which are potentially thrombogenic. Therefore, we apply this method for evaluating the blood coagulation process spiked with fibrinogen.

## Results

**Figure 1:**
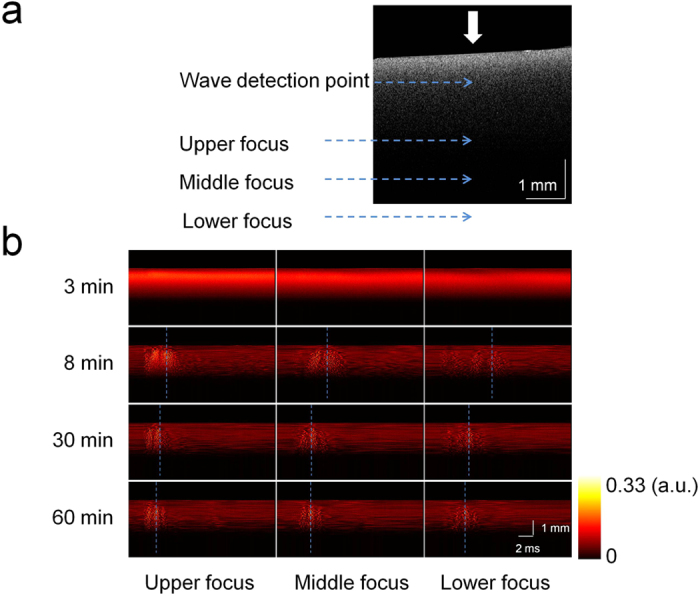
OCT and Doppler variance images for the blood coagulation measurement. (**a**) The OCT image at the beginning of the measurement. The ARF focus is moved downwards by a mechanical lifting stage at a step of 1mm for each measurement and the OCT M-scan is applied at the position indicated by the white arrow. (**b**) The Doppler variance images at four typical time points successively using three ARF focus positions. In the process of blood coagulation, the wave occurrence time of the shear wave becomes shorter, indicating a faster shear wave velocity using each of the ARF focus positions.

First, we investigate the feasibility of the ARFOE-OCE for measuring the shear modulus in the clotting blood. Time-course coagulation of the citrated porcine whole blood is investigated. Figure 1a shows the OCT image in a B-mode at 3 min following the recalcification of blood coagulation. For the shear wave detection, 43 M-scans are captured at the position indicated by the white arrow in Fig. 1a using each of three ARF excitation positions with a spacing of 1 mm. Figure 1b and Supplementary Videos 1,2–3 shows the Doppler variance images at different points successively using three ARF focus positions. At 3 min, no obvious vibration is detected; thus, no shear wave propagates to the OCT imaging area. After 8 min, the transverse vibration of the blood sample is detectable, which is generated by a shear wave propagating from the ARF focus. In the process of blood coagulation, the wave occurrence time becomes shorter, indicating a faster shear wave velocity using each of the ARF focus positions.

**Figure 2:**
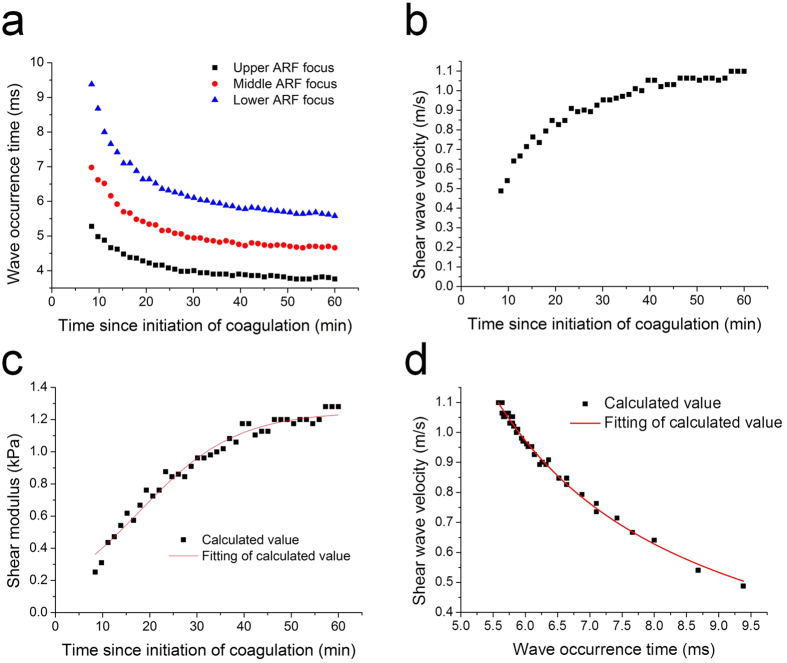
Analysis of the shear wave during blood coagulation. (**a**) The measured occurrence time of the shear wave at the wave detection point. Three ARF focus positions are applied for the measurement. (**b**) The calculated shear wave velocity during blood coagulation. The increase of the shear wave velocity indicates the blood clot becoming stiffer. (**c**) The shear modulus during blood coagulation. The shear modulus increases rapidly during the early stage of blood coagulation and becomes stable after 30 min. (**d**) Relation between the shear wave velocity and the shear wave occurrence time at the wave detection point with the lower ARF focus excitation.

We determine the reaction time by detecting the vibration occurrence after the Doppler variance value is 0.025 a.u. larger than the background for the detected peak. The measured occurrence time of the shear wave is shown in Fig. 2a. When the ARF focus is move downward at a spacing of 1 mm (that is, the ARF focus is moved away from the wave detection point), the wave occurrence time is delayed. The average shear wave velocity can be calculated by the distance of two ARF focus positions and the time difference of wave occurrence. The calculated shear wave velocities during 60 min are shown in Fig. 2b, which are measured by the application of the upper ARF focus and lower ARF focus. The density of the porcine blood clot changes slightly during the measurement so an average density *ρ* of 1060 kg/m^3^ is used in Eq. 1. The shear moduli during porcine whole blood coagulation are calculated by Eq. 1, as shown in Fig. 2c. The shear modulus increases rapidly during the early stage of blood coagulation and gradually reaches a plateau after 30 min. The shear modulus of the clotted blood at 46 min is 1.20 kPa from Fig. 2c, which is close to the value 1.39 kPa measured by the MTS Synergie 100 mechanical test. Figure 2d reveals a relation between the shear wave velocity *V* and the occurrence time 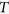 of the shear wave at the wave detection point with the lower ARF focus excitation, which can also be described by the following equation:


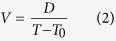


where *D* is the distance between the wave detection point and the ARF focus, and *T*_*0*_ is the occurrence time of the shear wave at the ARF focus. The fitting of the data in Fig. 2d using Eq. 2 reveals the distance *D* and the occurrence time *T*_*0*_; thus, the shear wave velocity can also be calculated by occurrence time 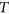 of the shear wave using Eq. 2.

In order to quantitatively characterize the elastic changes during blood coagulation, we developed coagulation metrics including reaction time describing the earliest time when the shear wave is detectable, clot formation kinetics describing the clot formation rate, and maximum shear modulus describing the maximum clot firmness. According to a previous study on elastic dynamics^7^, the change of the elastic modulus during blood coagulation can be modeled approximately by the logistic function, which is described by the following equation:


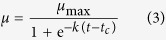


where 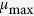 is the maximum shear modulus, 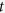 is the time since initiation of coagulation, 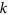 is the clot formation kinetics describing the clot formation rate, and 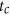 is time at the sigmoid’s midpoint.

Once blood starts to clot, fibrin strands are formed, progressively increasing the firmness of the blood. Figure 2c is presented as an elastic property tracing of clot formation, providing the information on clot formation kinetics and strength. Three distinct stages of the sample can be identified during coagulation. During the first stage, the blood is in a liquid state: there are no shear waves detectable. During the second stage, the blood progressively turns into a gel state because of the transformation of fibrinogen into fibrin through a coagulation cascade reaction. In this stage, the propagation velocity of the shear wave and, thus, the shear modulus, increases markedly with increasing firmness. In the third stage, the propagation velocity of the shear wave and, thus, the shear modulus, changes to a small extent with an approximate horizontal asymptote, corresponding to the stability and maximum firmness of the clot. Therefore, reaction time is defined as the time from the start of a sample run until the first detectable level of the shear wave in the Doppler variance image, similar to PT and aPTT^3^,^4^. Clot formation kinetics 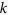 is defined as the clot formation rate and determined by Eq. 3 from the point of clot initiation to the peak strength of the clot. Maximum shear modulus represents the maximum clot firmness. This study demonstrates that the ARFOE-OCE is a feasible method for quantitatively analyzing the dynamic coagulation progress and status of whole blood.

**Figure 3:**
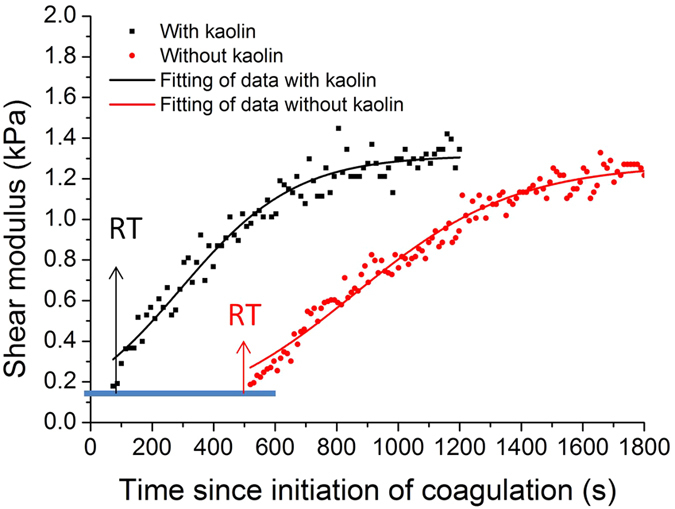
Comparison of shear modulus change with and without the kaolin during porcine whole blood coagulation. The reaction time is significantly different between the sample with kaolin and the sample without kaolin. The presence of kaolin results in an earlier reaction time.

When thromboelastometry assay is performed, kaolin may be used as the factor XII (FXII) activator in order to facilitate the clot formation^3^,^4^. Therefore, the capability of ARFOE-OCE for characterizing the different coagulation process activated by kaolin is evaluated and shown in Fig. 3. A saline solution is added to citrated porcine whole blood without kaolin as the control group (n = 3) and with kaolin as the treatment group (n = 3). There is a significant difference between the two groups of blood samples in the reaction time, and the presence of kaolin results in an earlier reaction time. The values of reaction time, clot formation kinetics and maximum shear modulus for two groups of blood samples are presented in Table 1. The presence of kaolin significantly influences the reaction time (P < 0.01) but not the clot formation rate (P > 0.05) or the peak firmness of clot (P > 0.05).

**Figure 4:**
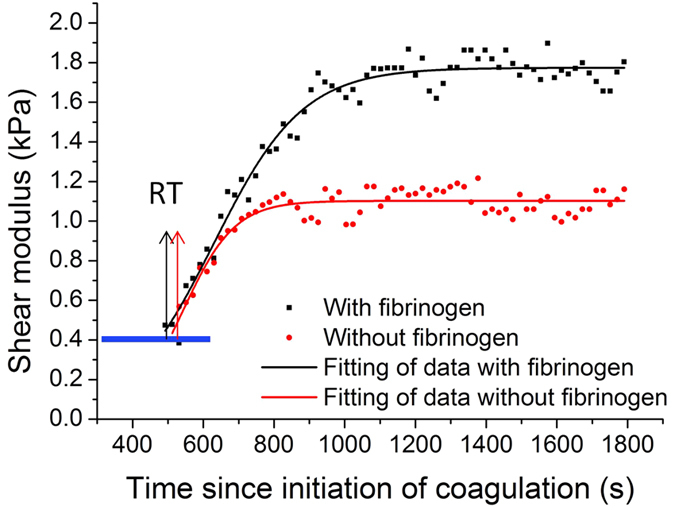
Comparison of shear modulus change with and without the fibrinogen during porcine whole blood coagulation. The additional fibrinogen changes the clot formation rate and increases the clot stiffness. No significant difference in the reaction time is measured between the control group and group spiked with fibrinogen.

The capability of ARFOE-OCE for characterizing the different coagulation process spiked by fibrinogen is evaluated and shown in Fig. 4. The tests are performed using porcine whole blood without fibrinogen (n = 3) and porcine whole blood spiked with fibrinogen (n = 3). The addition of fibrinogen does not significantly affect the reaction time (P > 0.05) but significant differences are observed between the control group and blood samples with additional 4 g/L fibrinogen in the clot formation kinetics (P < 0.01) and maximum shear modulus (P < 0.01), respectively. The values of reaction time, clot formation kinetics and maximum shear modulus for two groups of blood samples are presented in Table 2. The presence of additional fibrinogen significantly changes the clot formation rate and increases the peak firmness of clot.

To verify the method, we perform kaolin activating and fibrinogen spiking experiments by continuously recording the shear modulus during clot formation. The reaction time is significantly shortened after intrinsic activation with Kaolin, similar to the results from thromboelastometry measurement^4^. Great changes in clot formation rate and clot firmness are evident after addition of fibrinogen. The results are in agreement with the measurement by thromboelastometry^16^. Similar to the thromboelastometry measurement, the changes in maximum shear modulus measured by ARFOE-OCE should not be considered as a measurement of fibrinogen concentration, but a measurement of mechanical properties of the fibrin clot.

## Discussion

The novel approach described here represents the first elastic coagulation test method capable of measuring whole blood coagulation metrics, such as reaction time, clot formation kinetics, and maximum shear modulus, by measuring the propagation velocity of the shear wave. The foundation of our approach is our new ARFOE-OCE incorporating the Doppler variance method capable of high-speed and high-sensitivity functional imaging: thus producing non-contact and rapid measurements of blood coagulation in real-time without subsequent calibration. We have tested three samples for each group in this study. Although large sample size test may be required for the clinical validation, our results have demonstrated the capability of this method for the measurement of the whole blood coagulation metrics.

Using our method, the clot development can be visually displayed in real-time as the shear wave propagates, and the blood clot elasticity can be simply and accurately measured. Blood clot elasticity is a potentially powerful metric for predicting the risk associated with the presence of blood clots. Abnormal clot elasticity is associated with myocardial infarction, coronary atherothrombosis, ischemic stroke, venous thromboembolism, and diabetes. The importance of clot elastic modulus is evidenced by the growing use of thromboelastometry devices^3^,^4^. The coagulation metrics developed by our method are similar to TEG^R^ measurement, i.e., the time until initial fibrin formation (TEG^R^, reaction time; ROTEM^R^, clotting time), the kinetics of fibrin formation and clot development (TEG^R^, kinetics and α angle; ROTEM^R^, clot formation time and α angle), and the ultimate strength and stability of the fibrin clot (TEG^R^, maximum amplitude; ROTEM^R^, maximum clot firmness)^3^,^4^. More than 3000 publications are related to the use of thromboelastometry, in both research and clinical/perioperative settings. However, thromboelastometry devices generate output by transducing changes in the viscoelastic strength of clotting blood to which a constant rotational force is applied. The rotation of the pin of TEG^R^/ROTEM^R^ begins to be impaired after fibrin-platelet bonding has linked the cup and pin together. Thus, the output is directly related to the strength of the formed clot. Unlike thromboelastometry techniques, the output of ARFOE-OCE is sensitive to elasticity and monitors elasticity changes that occur during initiation of coagulation and clot development. Therefore, the approach has the advantage of being an OCE Doppler variance method that has the potential for high accuracy. The high sensitivity of the OCT system provides typically microstrain sensitivity. Thus, our approach may overcome several limitations of both routine coagulation tests and thromboelastometry measurement.

Because of the noninvasive nature, this technology also has the potential to perform *in vivo* imaging. This technology will have significant impacts in clinical researches, disease diagnoses and treatment management for bleeding patients in a variety of clinical situations, assessment of hypo- and hyper-coagulable states, and monitoring of pharmacological treatment with anti- and procoagulant drugs.

## Methods

### ARFOE-OCE setup

**Figure 5:**
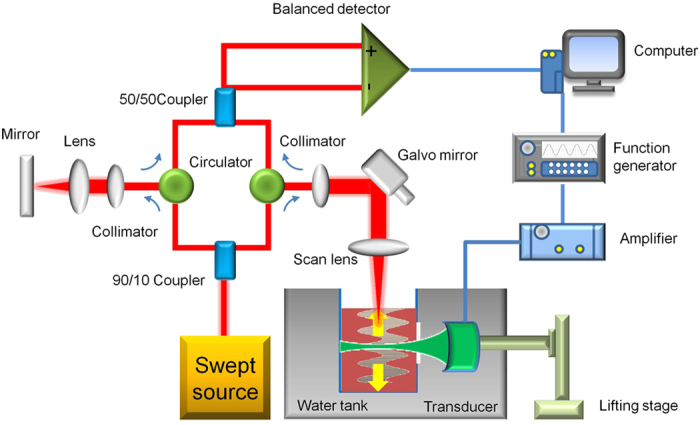
The schematic of the ARFOE-OCE system, including the swept source OCT unit and ultrasonic excitation unit. The vibration perpendicular to the OCT detection direction is detected by a Doppler variance method, and the shear wave propagating parallel to the OCT detection direction is measured by M-scans. The blood is filled to a box with a window of plastic wrap for the ultrasound wave passing. The box and the transducer are immersed in water for impedance matching of the ultrasound wave into the blood sample. Only the container contacts with the blood sample.

The experimental setup for the ARFOE-OCE is shown in Fig. 5. For ARF generation, an ultrasonic transducer with a resonant frequency of 4.5 MHz is driven by the amplified sine wave at 160 V peak-to-peak. The blood is filled to a box with a window of plastic wrap for the ultrasound wave passing. The box and the transducer are immersed in water for impedance matching of the ultrasound wave into the blood sample. The OCT system is based on a swept source with a central wavelength of 1310 nm and an A-line speed of 50 kHz. The light from the laser source is split into the sample arm and the reference arm with a power of 90% and 10%, respectively. For shear wave detection, 1000 A-lines at a rate of 50 kHz, corresponding to total time of 20.0 ms, are involved in one OCT M-scan, and the ARF excitation is applied between the 101st A-line to the 150th A-line corresponding to 1.0 ms emission. In this system, the ARF-induced vibration is perpendicular to the OCT beam, and the shear wave propagates along the OCT beam. Only the container contacts with the blood sample. The acoustic excitation system and optical detection system will not contact the blood sample.

### Image acquisition and analysis

The fresh citrated whole blood is placed in a container opened with a thin-film window through which the ultrasound can pass. As whole blood is a highly scattering media and the OCT penetration depth is shallow in blood^17^, it is difficult to visualize the shear wave propagation from the ARF focus to the sample surface. We use an alternative method to calculate the shear wave velocity, which is to measure the time delay of the shear wave propagating to the same depth after moving the ARF focus downwards to a known step by a mechanical lifting stage.

### Feasibility test

Fresh porcine blood is anticoagulated with sodium citrate (Sierra for medical science, USA). To activate coagulation, the blood sample of 9.0 mL is recalcified by adding 1.0 mL calcium chloride (0.2 M). After 10 gentle vial inversions, the mixed whole blood is immediately loaded into the blood container for the ARFOE-OCE dynamic measurements until 60 min.

### Kaolin effect test

A saline solution of 360 μL is added to 9 mL citrated porcine whole blood (Sierra for medical science, USA) without kaolin as the control group (n = 3) and with 20 g/L kaolin as the treatment group (n = 3). The mixtures are then triggered to clot by adding 530 μL calcium chloride (0.2 M). The shear wave is measured for 20 min during the coagulation of the blood with kaolin and for 30 min during the coagulation of the blood without kaolin which is enough time for the shear modulus to reach the plateau.

### Fibrinogen effect test

Fibrinogen solution (50 g/L) is prepared from fibrinogen powder (Sigma, USA) and saline and added to 9 mL citrated porcine whole blood (Sierra for medical science, USA), to increase fibrinogen level with 4 g/L from the initial concentration. One aliquot is mixed with saline as a control (n = 3). The mixtures are then triggered to clot by adding 530 μL calcium chloride (0.2 M). ARFOE-OCE tests are performed using native whole blood (n = 3) and whole blood spiked with fibrinogen (n = 3). The shear wave is measured for 30 min during the coagulation of the blood which is enough time for the shear modulus to reach the plateau.

## Additional Information

**How to cite this article**: Xu, X. *et al.* Dynamic and quantitative assessment of blood coagulation using optical coherence elastography. *Sci. Rep.* **6**, 24294; doi: 10.1038/srep24294 (2016).

## Supplementary Material

Supplementary Information

Supplementary Video 1

Supplementary Video 2

Supplementary Video 3

## Figures and Tables

**Table 1:** Coagulation metrics for kaolin effect.

Sample type	Reaction Time		Maximum shear modulus		Clot formation kinetics	
	Mean ± SD (s)	P-value	Mean ± SD (kPa)	P-value	Mean ± SD (1/s)	P-value
Group without kaolin	472 ± 50	P < 0.01	1.36 ± 0.08	P > 0.05	0.0038 ± 0.0005	P > 0.05
Group with kaolin	121 ± 41		1.25 ± 0.16		0.0049 ± 0.0007	

A saline solution is added to citrated porcine whole blood without kaolin as the control group (n = 3) and with kaolin as the treatment group (n = 3). The presence of kaolin significantly influences the reaction time but not the clot formation rate or the peak firmness of clot. P-values are calculated by the T-test.

**Table 2:** Coagulation metrics for fibrinogen effect.

Sample type	Reaction Time		Maximum shear modulus		Clot formation kinetics	
	Mean ± SD (s)	P-value	Mean ± SD (kPa)	P-value	Mean ± SD (1/s)	P-value
Group without fibrinogen	519 ± 30	P > 0.05	1.17 ± 0.07	P < 0.01	0.0135 ± 0.0009	P < 0.01
Group with fibrinogen	538 ± 49		1.69 ± 0.09		0.0078 ± 0.0001	

ARFOE-OCE tests are performed using native whole blood (n = 3) and whole blood spiked with fibrinogen (n = 3). The presence of additional fibrinogen significantly changes the clot formation rate and increases the peak firmness of clot. P-values are calculated by the T-test.
