# Supplementary material for: Dynamic and quantitative assessment of blood coagulation using optical coherence elastography
Source: Sci Rep. 2016 Apr 19;6:24294. doi: 10.1038/srep24294 (PMC4836302; doi:10.1038/srep24294)
Supplement: Supplementary Information [file srep24294-s1.doc]

**Dynamic and quantitative assessment of** **blood** **coagulation using** **optical coherence elastography**

Xiangqun Xu,^1,2*^ Jiang Zhu,^2*^ and Zhongping Chen^2,3^

^1^College of Life Sciences, Zhejiang Sci-Tech University, Hangzhou, Zhejiang 310018, China

^2^Beckman Laser Institute, University of California, Irvine, Irvine, California 92612, USA

^3^ Department of Biomedical Engineering, University of California, Irvine, Irvine, California 92697, USA

Correspondence and requests for materials should be addressed to X.X. (**xuxiangqun@zstu.edu.cn**) or Z.C. (z2chen@uci.edu)

* These authors contributed equally to this work.

**Supplementary Video 1.** Doppler variance images during the blood coagulation measurement using shallow ultrasonic focus.

**Supplementary Video 2.** Doppler variance images during the blood coagulation measurement using middle ultrasonic focus.

**Supplementary Video 3.** Doppler variance images during the blood coagulation measurement using deep ultrasonic focus.
